# *NDUFAF6*-Related Leigh Syndrome Caused by Rare Pathogenic Variants: A Case Report and the Focused Review of Literature

**DOI:** 10.3389/fped.2022.812408

**Published:** 2022-05-18

**Authors:** Jaewon Kim, Jaewoong Lee, Dae-Hyun Jang

**Affiliations:** ^1^Department of Rehabilitation Medicine, Incheon St. Mary’s Hospital, College of Medicine, The Catholic University of Korea, Seoul, South Korea; ^2^Department of Laboratory Medicine, Incheon St. Mary’s Hospital, College of Medicine, The Catholic University of Korea, Seoul, South Korea

**Keywords:** Leigh syndrome, mitochondrial disease, *NDUFAF6*, complex I deficiency, neurodegenerative disorder

## Abstract

Leigh syndrome is a neurodegenerative disorder that presents with fluctuation and stepwise deterioration, such as neurodevelopmental delay and regression, dysarthria, dysphagia, hypotonia, dystonia, tremor, spasticity, epilepsy, and respiratory problems. The syndrome characteristically presents symmetric necrotizing lesions in the basal ganglia, brainstem, cerebellum, thalamus, and spinal cord on cranial magnetic resonance imaging. To date, more than 85 genes are known to be associated with Leigh syndrome. Here, we present a rare case of a child who developed Leigh syndrome due to pathogenic variants of *NDUFAF6*, which encodes an assembly factor of complex I, a respiratory chain subunit. A targeted next-generation sequencing analysis related to mitochondrial disease revealed a missense variant (NM_152416.4:c.371T > C; p.Ile124Thr) and a frameshift variant (NM_152416.4:c.233_242del; p.Leu78GInfs*10) inherited biparentally. The proband underwent physical therapy and nutrient cocktail therapy, but his physical impairment gradually worsened.

## Introduction

Mitochondrial disease is a common inherited metabolic disorder that is clinically and genetically heterogeneous and occurs in approximately 10–25 cases per 100,000 individuals, many of which are childhood-onset usually by the age of 3 years (1). Mitochondria are present in all cells except erythrocytes and, given that mitochondria are responsible for oxidative phosphorylation and ATP synthesis, mitochondrial disease exhibits diverse phenotypes depending on the affected organs. Mitochondrial dysfunction leads to multiple system disorder, manifesting a progressive course with high morbidity and mortality (2). Mitochondrial disease is a genetic disorder resulting from pathogenic variants of mitochondrial DNA (mtDNA) and nuclear DNA (nDNA) associated with mitochondrial function. The mtDNA consists of 37 genes and encodes 13 proteins, and over 1,000 nuclear genes encode proteins related to mitochondrial function (3).

The most common mitochondrial disease that occurs during childhood is Leigh syndrome, with a prevalence of approximately 2.5 cases per 100,000 individuals (4). Most cases of Leigh syndrome occur in childhood and rarely in adolescence or early adulthood. Leigh syndrome is a neurodegenerative disorder, with characteristic symmetric, necrotizing lesions in the basal ganglia, brainstem, cerebellum, thalamus, and spinal cord on cranial magnetic resonance imaging (MRI). The phenotypes of Leigh syndrome include neurological symptoms showing fluctuation and stepwise deterioration, such as neurodevelopmental delay and regression, dysarthria, dysphagia, hypotonia, dystonia, tremor, spasticity, epilepsy, and respiratory problems. Other non-neurologic symptoms include cardiac abnormalities, hepatorenal anomalies, anemia, and gastrointestinal symptoms such as constipation and vomiting (5). More than 85 genes are related to Leigh syndrome including mtDNA and nDNA, and genes related to complex I (NADH: ubiquinone oxidoreductase) deficiency are the most common causes. Approximately 75–80% of cases of Leigh syndrome are caused by pathogenic nDNA variants and more than half of the nDNA genes are related to complex I. *NDUFAF6* (NADH: ubiquinone oxidoreductase complex assembly factor 6) is one of them, and *NDUFAF6* is one of the major genes that cause reduced complex I activity by mitochondrial respiratory chain complex defect. Biallelic missense variants of *NDUFAF6* are known to cause Leigh syndrome.

Here, we present a rare case of a child who developed Leigh syndrome due to pathogenic variants of *NDUFAF6* on chromosome 8, which encodes an assembly factor of complex I, a respiratory chain subunit. A targeted next-generation sequencing study (NGS) enabled rapid genetic confirmation and avoided invasive diagnostic procedures such as skeletal muscle biopsy and skin fibroblast cell culture.

## Case Presentation

A 56-month-old boy was referred to a rehabilitation clinic due to left hand clumsiness and frequent falling. The symptoms gradually began around the age of 3 years and slowly progressed. The patient had been born after an uncomplicated 40-week gestation with a birth weight of 3.07 kg. There were no perinatal problems and no specific family history of interest. There was no psychomotor developmental abnormalities prior to the onset of symptoms. The patient was brought to the primary clinic at 55 months due to language developmental delay, with simplified sentences and inaccurate pronunciation. In the evaluation, the patient’s overall comprehension and language expression level was measured around 56–60 months, and his consonant accuracy was evaluated at approximately 87% in the pronunciation intelligibility assessment. The patient also presented with weakened tongue movement, simplification of middle consonants, and simplification of liquid consonants. In the psychoeducational profile–revised (PEP-R) evaluation, the overall developmental level was 64–65 months, and the other domains were above the normal ranges, except for fine motor function, which was below the average of 52–54 months. During the examination, the patient presented poor bimanual manipulation, with left hand clumsiness and slight weakness of the right ankle dorsiflexion; the patient was referred to a tertiary medical center.

### Clinical Assessment

During the physical examination, the patient presented with dystonic and ataxic movements on the left side and clumsy movements of both hands. Weakness in ankle dorsiflexion involving both sides and spastic hypertonia in the left upper and lower extremities were observed. The strength of both ankle dorsiflexors was 3/5, and the strength of the other muscles was 5/5. The patient had difficulty jumping on both feet and stair-climbing and descending. Poor balance with one-leg standing with eyes open was also observed. The patient could independently walk but had tip-toeing and limping gait patterns. In the functional assessment, the balance evaluation using the Berg balance scale (BBS) revealed a poor standing balance of 36/56. The total score on the gross motor function measure-88 (GMFM-88) was 81.82%. The Beery–Buktenica developmental test of visual motor integration (6th edition) revealed a normal range of visual motor function of 99 (47%) with impaired motor coordination ability of 73 (4%).

### Neuroimaging and Laboratory Tests

**FIGURE 1:**
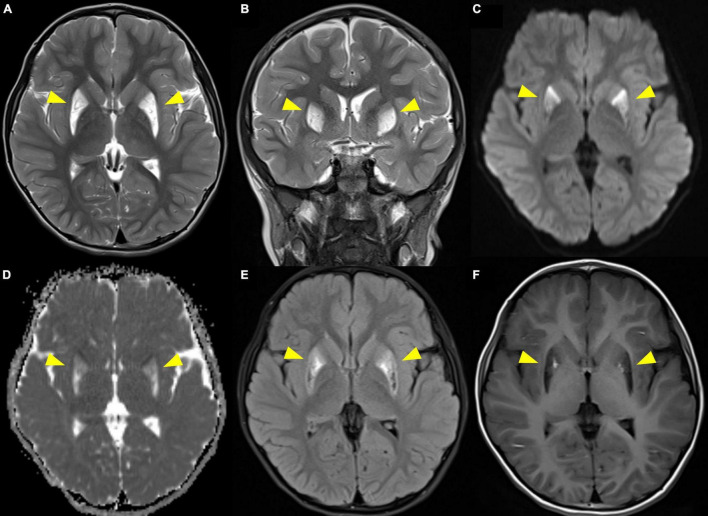
Brain MRI showing high signal intensity on T2-weighted imaging (**A**. axial, **B**. coronal) and diffusion-weighted imaging **(C)**, apparent diffusion coefficient **(D)**, and fluid-attenuated inversion recovery **(E)** images with low signal intensity on T1-weighted imaging **(F)** in the bilateral putamen. Arrowheads indicate the lesions.

**FIGURE 2:**
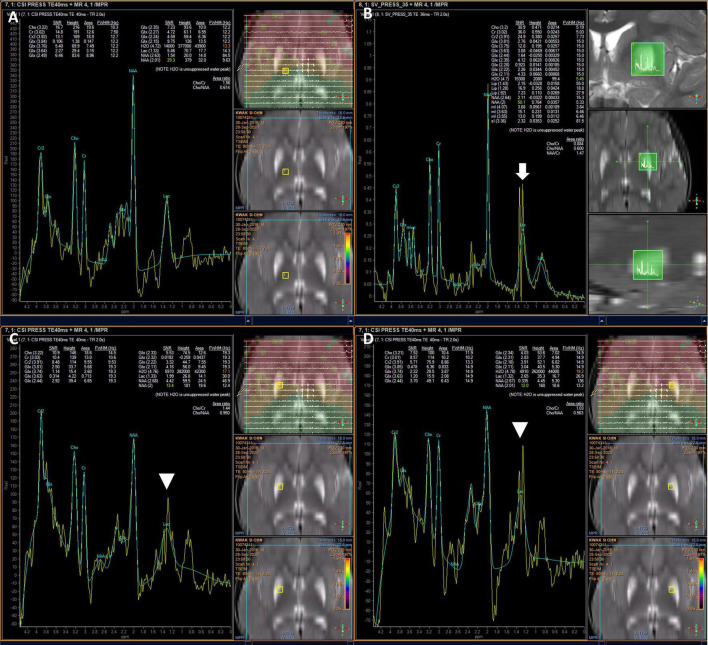
Brain MR spectroscopy showing increased lipid and lactate in the lesions. Normal patterns outside lesions **(A)**, elevated lipids (white arrow) **(B)**, and elevated lactate in both putamen (arrowhead) **(C,D)**.

Due to a suspected brain pathology, an MRI of the head was performed, revealing abnormal signals in the bilateral putamen, with slight volume reduction and partial necrosis. The MRI revealed high signal intensity on T2- and diffusion-weighted images, an apparent diffusion coefficient (ADC), and fluid-attenuated inversion recovery (FLAIR) images with low signal intensity on T1-weighted images in the bilateral putamen (Figure 1). MR angiography showed normal cerebral vessels. To differentiate diseases that could show lesions of symmetric basal ganglia, we also performed MR spectroscopy, detecting normal N-acetylaspartate, choline, and creatine levels, and increased lipid and lactate levels (Figure 2). The high lactate peak would indicate acute mitochondrial dysfunction and with clinical features, we determined that there was a possibility of mitochondrial encephalopathy, especially Leigh syndrome. The laboratory test revealed a lactic acid level of 2.45 mmol/L (range, 0.50–2.20 mmol/L) and a creatine phosphokinase level of 287 U/L (range, 0–250 U/L). In the urine organic acid test, a slight increase was observed in tiglylglycine levels to 0.4 mmol/mol creatine (range, 0–0.2 mmol/mol creatine). The other laboratory test results, including serum aspartate aminotransferase, alanine aminotransferase, amino acid analysis, and acylcarnitine were all within normal ranges.

### Cytogenetic and Molecular Analyses

**FIGURE 3:**
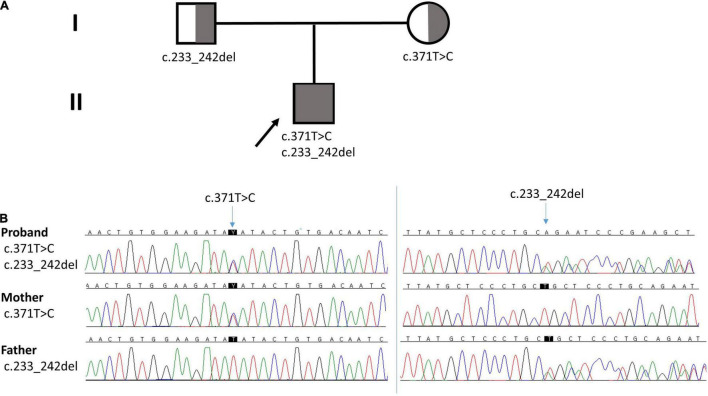
**(A)** Pedigree of the family. Arrow indicates the proband. **(B)** Chromatographs of *NDUFAF6*. The proband had a missense variant (c.371T > C) from the mother and a frameshift variant (c.233_242del) from the father.

We performed a mitochondrial DNA sequencing test and targeted NGS analysis of a multi-gene panel related to the mitochondrial disease. Informed consent from the patient’s guardian was collected. Libraries were prepared with the Illumina™ Truseq DNA kit, and sequencing was performed using the Illumina™ Nextseq platform. The whole mtDNA test revealed a normal mtDNA sequence. The NGS test revealed two heterozygous variants of *NDUFAF6* in the different exons, identifying a missense variant (NM_152416.4:c.371T > C; p.Ile124Thr) and a frameshift variant (NM_152416.4:c.233_242del; p.Leu78GInfs*10), which were validated by Sanger sequencing. No other significant variants were found. The missense variant c.371T > C has been previously reported to cause Leigh syndrome, while the c.233_242del variant has not been reported (6–8). The missense variant c.371T > C was classified as a likely pathogenic variant and the frameshift variant (c.233_242del) was classified as a pathogenic variant by American College of Medical Genetics and Genomics (ACMG) Classification Standards and Guidelines for Genetic Variations. *In silico* prediction for the missense variant, SIFT predicted as deleterious (score: 0.05), PolyPhen-2 as benign (score 0.255), and MutationTaster as disease causing (probability: 0.99). For the frameshift variant, MutationTaster predicted as disease causing (probability: 1). To identify these compound heterozygous variants, we performed targeted gene sequencing of the parents, which revealed that the c.371T > C variant was inherited from the mother while the c.233_242del variant was inherited from the father (Figure 3). Finally, the proband was diagnosed with Leigh syndrome caused by biallelic pathogenic variants of *NDUFAF6*, inherited biparentally.

### Clinical Progressions

After the diagnosis, the patient continued physiotherapy and occupational therapy while undergoing nutrient cocktail therapy. He was prescribed 10 mg of biotin, 500 mg of thiamine, 100 mg of Coenzyme Q10, and 100 mg of L-carnitine per day. However, worsening and improvement of spastic dystonia and balance impairment were repeated and his gait instability gradually worsened.

Five months after the first visit and at the age of 61 months, the patient’s BBS dropped from 36/56 to 11/56, and his GMFM-88 score worsened from 81.82 to 58.33%. He required support from two hands for walking and for transitioning from sitting to standing. He was unable to maintain an upright sitting or standing position and showed poor sitting balance. A symmetric tonic neck reflex appeared, and a hand-foot crawling pattern was observed.

## Discussion

We have reported a proband with biallelic pathogenic variants, c.371T > C and c.233_242del in *NDUFAF6*, who showed the typical phenotype of Leigh syndrome. The proband presented Leigh syndrome-appropriate clinical features, brain MRI and MR spectroscopy findings, and genetic confirmation. However, the serum biomarkers showing oxidative phosphorylation impairment such as lactate, pyruvate, creatine kinase, amino acid, acylcarnitine, and organic acid revealed no disease-specific findings. The patient underwent rehabilitation therapy and nutrient cocktail therapy, but his physical impairment rapidly worsened.

**FIGURE 4:**
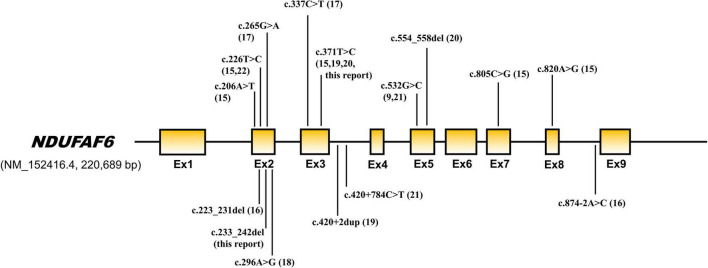
Genetic structure of *NDUFAF6*. Pathogenic variants previously reported are indicated by arrows.

NDUFAF6 is an assembly factor of NADH-ubiquinone oxidoreductase (complex 1) and plays an important role in complex 1 maturation and activity. NDUFAF6 is a 38 kDa protein, consists of 333 amino acids, and is involved in the assembly of complex I in early stages, playing a role in regulating and stabilizing the complex I subunit MT-ND1. Defects in NDUFAF6 isoform 1 can disrupt complex I assembly by causing rapid proteolysis of the newly formed ND1 subunit, adversely affecting mitochondrial function (9, 10). Biallelic pathogenic variants cause Leigh syndrome, and 16 variants have been reported in 20 patients to date (Table 1 and Figure 4). Among them, the most variants have been reported in exon 2, and 2 variants have also been reported in exons 3 and 5. All children with *NDUFAF6*-related Leigh syndrome show necrotic lesions in the symmetric basal ganglia, dystonia, and neurological regression. Characteristically, *NDUFAF6*-related Leigh syndrome tends to show normal or slight higher serum lactate, and our proband also showed lactate close to the normal range, revealing little diagnostic value for Leigh syndrome.

**TABLE 1:** Variants and clinical features of patients with *NDUFAF6*-related Leigh syndrome.

	This case	Johnstone et al. (7)	Baide-Mairena et al. (8)	Bianciardi et al. (12) Catania et al. (18)	Fang et al. (19)	Pagliarini et al. (20)	Martikainen et al. (21)
Number of proband	1	1	3	4	1	2	2
Variants	c.371T > C c. 233_242del	c.371 T > C c.420 + 2_420 + 3insTA	c.371 T > C c.554_558del	c.532G > C c.420 + 784C > T	c.337C > T c.265G > A	c.296A > G*	c.226T > C*
Age of onset	3 years	4 years	17 months, 30 months, 30 months	21 months, 12 months, 3.5 years, 5 years	4 years	7 months, 10 months	1 year
Clinical features	Dysarthria, poor bimanual manipulation, dystonic and ataxic movement, poor balance, neurologic regression	Tiptoeing, increasingly severe focal hand dystonia which eventually became generalized dystonia, dysarthria, ataxic gait	Gait loss, speech difficulties, neurological deterioration, generalized dystonia, dysphagia, dysarthria	Ataxic gait, fine tremor, drooling, dysarthria, dysmetria, tremor, hypertonia, dystonic movement, fine motor difficulty, cognition preservation	Movement disorder, abnormal gait, exercise intolerance, weakness, difficulty swallowing, increased muscle tension	Focal right-hand seizures, decreased movement and strength, ataxia, and evolving rigidity	Generalized dystonia, stepwise deterioration
Lesions with abnormal signals on MRI	Bilateral putamen	Bilateral putamen	Bilateral caudate and putamen	Bilateral caudate and putamen	Bilateral basal ganglia, and gradually expand to centrum semiovale	Consistent with LS	Bilateral caudate, putamen, parietal cerebral white matter, dorsal pons
Serum lactate (mmol/L) (range, 0.50–2.20)	2.45 mmol/L	Normal	Normal	Slightly increased, normal	Normal	High	N/A

*Homozygous; Variants for which clinical features were not described [Khoda et al. (6)—four patients: c.226T > C, c.805C > G; c.206A > T, c.371T > C; c.371T > C, 805C > G; 820A > G*, Lee et al. (22)—two patients: c.265G > A, c.233_231del; c.874-2A > C, c.820A > G].

In a cohort study of 166 patients with Leigh syndrome, 8 had pathogenic variants of *NDUFAF6*. As a result of the analysis, mtDNA variants were associated with high mortality, and patients with *NDUFAF6* variants had better outcomes than those with other nDNA gene variants showing lower mortality and longer motor function preservation (11). However, not all patients with *NDUFAF6*-related Leigh syndrome show mild phenotypes, and the genotype does not appear to predict the prognosis sufficiently, given that there are patients with early disease onset with severe symptoms who have the same variants (12). Our proband showed normal psychomotor development with uneventful medical history until the age of 3, and symptoms developed gradually. At 55 months, he showed mild motor impairment, but from then on, he showed rapid deterioration. Since there is no report on long term functional prognosis in the *NDUFAF6*-related Leigh syndrome, additional reports will be helpful for prognosis prediction.

Mitochondrial disease is difficult to diagnose due to the diverse clinical features. Previously, tissue biopsy was often performed for diagnosis by histochemical and biochemical analysis. With the recent development of NGS technology, there have been numerous cases that relied on genetic tests. In addition, cases previously diagnosed with probable or uncertain mitochondrial disease have more frequently been genetically confirmed. As a result, overall diagnostic success has risen from less than 20% to more than 60% (1). In particular, given that 75–80% of cases of childhood-onset mitochondrial disease occur due to the pathogenic variant of nDNA, the NGS test increases the diagnosis success (13). In a study conducted in 2015, patients who were ultimately diagnosed with mitochondrial disease had met an average of 8.19 physicians before being confirmed, and 70% of the patients had undergone invasive muscle biopsy. Rapid diagnosis without invasive tests has been made possible with the advances in genetic diagnostic technology, including whole exome sequencing and whole genome sequencing (14).

Treatment for Leigh syndrome has no clear evidence, but cocktails of nutritional dietary supplements, such as antioxidants, vitamins, coenzyme Q10, and nitric acid precursors have been widely used (15). Given that Leigh syndrome is genetically heterogeneous, early intervention with tailored therapy according to the genotype rather than treatment according to the phenomenon will be an important treatment direction in the future. In terms of rehabilitation, exercise prescription including aerobic exercise training is often performed for patients with mitochondrial disease with gait disturbance, imbalance, frequent falling, fatigue, and exercise intolerance. In general, excessive physical activity is not recommended, and physiotherapy is contraindicated for patients with cardiac involvement. Physiotherapy is also not recommended for those with acute illness, severe fatigue, and a generally deconditioned status because of the expected excessive energy demand in addition to the body’s energy demands (16). However, there was a report that oxygen utilization, exercise tolerance, and quality of life were better for the group who continued exercise training than for the group who did not (17). Therefore, rehabilitation therapy should be determined individually. Our patient underwent rehabilitation therapy because his general condition was not poor and did not correspond to discontinuation; however, his physical function gradually deteriorated.

## Conclusion

We report a rare genetic disorder: *NDUFAF6*-related Leigh syndrome resulting from biallelic pathogenic variants, c.371T > C and c.233_242del in *NDUFAF6*. The proband showed typical Leigh syndrome features, and his physical impairment gradually worsened despite several therapies. This case report is distinguished from those previously reported in that we describe in detail the clinical features, including the developmental evaluation and clinical progressions.

## Data Availability Statement

The original contributions presented in the study are included in the article/supplementary material, further inquiries can be directed to the corresponding author.

## Ethics Statement

The studies involving human participants were reviewed and approved by the Institutional Review Board for Clinical Research at Incheon St. Mary’s Hospital. Written informed consent to participate in this study was provided by the participants’ legal guardian/next of kin.

## Author Contributions

JK: acquisition of data, analysis and interpretation of data, and writing. JL: analysis and interpretation of data. D-HJ: study concept and design, acquisition of data, analysis and interpretation of data, study supervision, and critical revision of manuscript for intellectual content. All authors contributed to the article and approved the submitted version.

## Conflict of Interest

The authors declare that the research was conducted in the absence of any commercial or financial relationships that could be construed as a potential conflict of interest.

## Publisher’s Note

All claims expressed in this article are solely those of the authors and do not necessarily represent those of their affiliated organizations, or those of the publisher, the editors and the reviewers. Any product that may be evaluated in this article, or claim that may be made by its manufacturer, is not guaranteed or endorsed by the publisher.
